# Fighting Bisphenol A-Induced Male Infertility: The Power of Antioxidants

**DOI:** 10.3390/antiox10020289

**Published:** 2021-02-15

**Authors:** Joana Santiago, Joana V. Silva, Manuel A. S. Santos, Margarida Fardilha

**Affiliations:** 1 Department of Medical Sciences, Institute of Biomedicine-iBiMED, University of Aveiro, 3810-193 Aveiro, Portugal; joanasantiago@ua.pt (J.S.); joanavieirasilva@ua.pt (J.V.S.); msantos@ua.pt (M.A.S.S.); 2 Institute for Innovation and Health Research (I3S), University of Porto, 4200-135 Porto, Portugal; 3 Unit for Multidisciplinary Research in Biomedicine, Institute of Biomedical Sciences Abel Salazar, University of Porto, 4050-313 Porto, Portugal

**Keywords:** Bisphenol A, endocrine disruptors, male infertility, oxidative stress, antioxidants, phytochemicals, medicinal plants

## Abstract

Bisphenol A (BPA), a well-known endocrine disruptor present in epoxy resins and polycarbonate plastics, negatively disturbs the male reproductive system affecting male fertility. In vivo studies showed that BPA exposure has deleterious effects on spermatogenesis by disturbing the hypothalamic–pituitary–gonadal axis and inducing oxidative stress in testis. This compound seems to disrupt hormone signalling even at low concentrations, modifying the levels of inhibin B, oestradiol, and testosterone. The adverse effects on seminal parameters are mainly supported by studies based on urinary BPA concentration, showing a negative association between BPA levels and sperm concentration, motility, and sperm DNA damage. Recent studies explored potential approaches to treat or prevent BPA-induced testicular toxicity and male infertility. Since the effect of BPA on testicular cells and spermatozoa is associated with an increased production of reactive oxygen species, most of the pharmacological approaches are based on the use of natural or synthetic antioxidants. In this review, we briefly describe the effects of BPA on male reproductive health and discuss the use of antioxidants to prevent or revert the BPA-induced toxicity and infertility in men.

## 1. Introduction

Environment and diet strongly influence spermatogenesis, having significant consequences on male fertility and reproductive potential. There was a rising concern about human exposure to endocrine-disrupting chemicals (EDCs) and their release into the environment [1,2]. An endocrine disruptor is defined by the World Health Organization as “an exogenous substance or mixture that alters the function(s) of the endocrine system and consequently causes adverse health effects in an intact organism, or its progeny, or (sub)populations” [3]. EDCs may act by mimicking the biological activity of an hormone (agonistic effect), blocking its activity by binding to the receptor without activating it (antagonistic effect) or interfering with the synthesis or elimination rates of the natural hormones, even at extremely low doses (picomolar to nanomolar) [4,5]. Indeed, an important feature of EDCs is their unusual dose–response dynamics (usually inverted-U or U-shaped curves), since low doses may in some cases exert more potent effects than higher doses [5,6]. This characteristic, called non-monotonic response, complicates the assessment of potential impacts of exposure and makes the use of a dose test to predict low-dose effects inappropriate [5]. Moreover, it is important to consider that environmental exposure usually involves EDC mixtures, whose constituents can act through a common mode or by several mechanisms of action which might crosstalk [4,6]. This combined effect may have an additive, synergistic or attenuative potential [4,6]. EDC exposure during foetal development infancy, childhood and puberty can have long-lasting health effects since at these moments, hormones strongly regulate the formation and maturation of organs. Early-life exposures have also been associated with developmental abnormalities and may increase the risk of several diseases later-in-life [4]. In adulthood, increasing incidences of several human reproductive disorders, such as testicular cancers and reduced sperm counts, may be partially attributed to an increased exposure to environmental EDCs that have estrogenic activity [7,8,9,10,11].

Bisphenol A (BPA) represents one of those environmental chemical pollutants that mimic the natural oestrogen 17-β-oestradiol (E2). Epidemiological data from US revealed that 90% of general population have detectable levels of BPA in urine [12,13]. Its widespread presence in several daily used products and its detection in several human tissues and body fluids (urine, blood, serum, amniotic fluid, and semen) [14,15,16] raised many concerns about its potential association with human disorders such as cancer, cardiovascular diseases, obesity, diabetes, and reproductive disorders [7,13,17]. Although BPA may be toxic for other organs, attention has been paid to its reproductive and endocrine disrupting effects [18,19,20]. The toxicity of BPA, especially at the reproductive level, results from its interaction with androgen and oestrogen receptors [21,22,23,24]. Although BPA is not an oxidizer itself, it leads to cellular changes usually manifested by lipid peroxidation (LPO) and free radicals production causing oxidative stress (OS) [21,22,23,24]. In the past decade, the use of antioxidants such as melatonin [25,26], vitamin C [27], N-acetylcysteine [28], coenzyme Q10 [29], and several plant extracts [30,31,32], to prevent and/or revert BPA-induced testicular toxicity started to be investigated. In this review, we briefly describe the effects of BPA on male reproductive health and discuss the use of antioxidants to prevent or revert the BPA-induced toxicity and infertility in men.

## 2. BPA: What Is This?

Bisphenol A (4,40-isopropylidenodi-phenol compound 2,2-bis (4-hydroxylphenyl)-propane) is a crystalline chemical compound widely used as a monomer in industry to produce plastic materials (polycarbonate, phenol, and epoxy resins), polyesters, and polyacrylate. During the past 50 years, this compound has often been used as an additive and/or antioxidant in polyvinyl chloride (PVC) production and processing, cosmetics and as a plastic softener [33]. Among many applications, this compound is present in several daily use products, such as containers to line food and beverage, plastic dishes, kitchen utensils, dental sealants and fillers, electronics (fridges, hair dryers, cell phones, computers) and thermal paper [34]. Due to its resiliency, flexibility, and durability, BPA has also been used in the manufacture of arms, safety equipment (helmets), and medical devices [34]. As a component of epoxy resins, BPA is also present on the internal coating of cans used in canned food [35]. The main route of exposure of BPA is dietary ingestion, since the exposure to temperatures higher than 70 °C and the reutilization of containers results in BPA leakage to food and beverage [36,37]. However, the risk of exposure through inhalation [38,39,40] and skin contact, especially through thermal paper [41,42,43], is also considerable.

After entering the organism, arround12% of BPA is metabolized in the liver by glucuronidation—BPA quickly binds to glucuronic acid by the liver enzyme uridine diphosphonate glucuronosyl transferase (UGT) producing BPA glucuronide (BPA-G) [16,44]. This process increases BPA water solubility with a consequent faster excretion in urine (half-life of elimination of 5.4–6.4 h), which means that humans exposed to oral doses of BPA ranging from 50 to 100 µg/kg body weight have less than 1% free BPA after 24 h [44]. The possible BPA bioaccumulation in the liver associated with its ingestion remains a topic of debate. However, contrary to dietary exposure, almost all BPA resulting from transdermal exposure avoids the liver metabolism, resulting in significantly higher concentrations of the unconjugated form (free BPA) in the bloodstream [45,46]. Considering that only free BPA has a biologically active role, the effects of transdermal exposure on human’s health represents a major concern. Currently, total urinary BPA (conjugated and unconjugated forms) is generally used as a biomarker of exposure to this chemical [47].

Considered an EDC, BPA disturbs the normal hormonal signalling resulting in adverse effects for the whole organism. In 1998, Gould [48] and Kuiper [49] showed that free BPA interacts with oestrogen receptor α (ERα), activating it in a manner distinct from the classical pattern observed in weak oestrogens, partial agonists and antagonists. Recently, it was reported that free BPA binds several nuclear receptors by (i) mimicking the action of endogenous steroids, (ii) maintaining the target molecule in active conformations and (iii) blocking the access of endogenous E2 to the receptor’s binding site by competition [18,50]. However, based on the available evidence, BPA has a very weak binding affinity to oestrogen receptor, being almost 10,000 times weaker than that of natural E2 [51]. Additionally, it may also bind to other receptors such as G protein-coupled oestrogen receptor 30 (GPR30/GPER1) [52,53], orphan nuclear oestrogen-related receptor gamma (ERR-γ) [54,55], androgen receptor (AR), peroxisome proliferator-activated receptor gamma (PPAR-γ), and thyroid hormone receptor (TR) [56]. The binding to these receptors may lead to other alterations in cells and tissues rather than endocrine disturbance.

Considering that several studies showed deleterious effects of BPA exposure, several BPA Product Regulations have been created. Regulation (EU) 10/2011 and its amendment Regulation (EU) 2018/213 banned in European Union the use of BPA in feeding bottles, plastic cups and packaging containing food intended to be used by infants and children younger than 3 years old; and introduced stricter limits on BPA in food contact materials [57,58]. Since 2020, REACH directives (Regulation (EC) No 1907/2006) mandates that thermal paper cannot contain a BPA concentration equal to or greater than 0.02% by weight. Moreover, several European countries adopted their own measures regarding BPA. For instance, Sweden (Regulation SFS 2012:991), Belgium (Act of 4 September 2012), and Denmark (Statutory Order No. 822) prohibited BPA in food contact materials for infants and children under the age of 3 years old; France (Law No. 2012-1442) forbidden BPA in all food packaging intended to be in direct contact with food. However, the recent results of the CLARITY (Consortium Linking Academic and Regulatory Insights on BPA Toxicity)-BPA study intensified the controversy around this topic. This study was conducted by a consortium of US government scientists and several academic research groups having two components—the core study [59] and 14 grantee studies [60]. The core study consisted in three groups of pregnant rats (control group, BPA-exposed and oestrogen exposed), in which the female rats and the offspring were exposed to different concentrations of BPA throughout their whole lifespan (continuous dose), or by “stop-dose“ [59,61,62]. Several tissues were examined (brain, heart, mammary gland, ovaries, prostate, testis, etc.) to determine if (a) the continuous exposure was directly relevant for human exposure and safety assessment, (b) the “stop-dose” exposure can be effectively used to investigate whether developmental exposure shows adverse effects later in life, and (c) the effects at low doses and/or non-monotonic dose–responses could be seen [61,63]. Overall, the results indicated that there was no evidence of non-monotonic dose–response, or relevant adverse effects of developmental exposure later in life [61]. The authors concluded that BPA is safe for consumers at typical consumer exposure levels. As the European Food Safety Authority (EFSA) started a re-evaluation of the safety of BPA for food contact applications in 2017 that will include the CLARITY-BPA study, it is possible that some policies may be updated.

## 3. BPA-Induced Alterations in Testicular Structure, Function, and Semen Parameters

In the past century, increasing attention has been paid to BPA effects on human’s health [64,65]. Since then, the associations between BPA levels and testicular toxicity, semen parameters, and overall male fertility have been extensively studied. Importantly, the severity of BPA impact on the male reproductive system depends on age, dose, mode, and duration of exposure [19,66]. In fact, methodological differences and distinct study populations can explain some of the contradictory results. In in vivo studies, BPA is typically administered in rodents orally. The doses usually range from 0.05–1 mg/kg/day for 30 days to 10 mg/kg/day during two weeks in mice. Rats were generally exposed to higher concentrations of BPA, ranging from 25 mg/kg/day during 60 days to 200 mg/kg/day for 10–30 days. Moreover, BPA and its metabolites have been measured in the plasma (<LLOQ (0.0435 µg/L)–7.23 µg/L; median 0.093 µg/L [67]), blood (0.19 ± 0.16 µg/L [68]), urine (1.66 ± 1.31 µg/L [68]), and seminal fluid (<LLOQ (0.0289 µg/L)–10.9 µg/L; median 0.085 µg/L [67]) in men. Based on new toxicological data and methodologies, the European Authorities adjusted the tolerable daily intake from 50 to 4 µg/kg/day, which may be revised soon according to the results of the CLARITY-BPA study [59].

**Figure 1:**
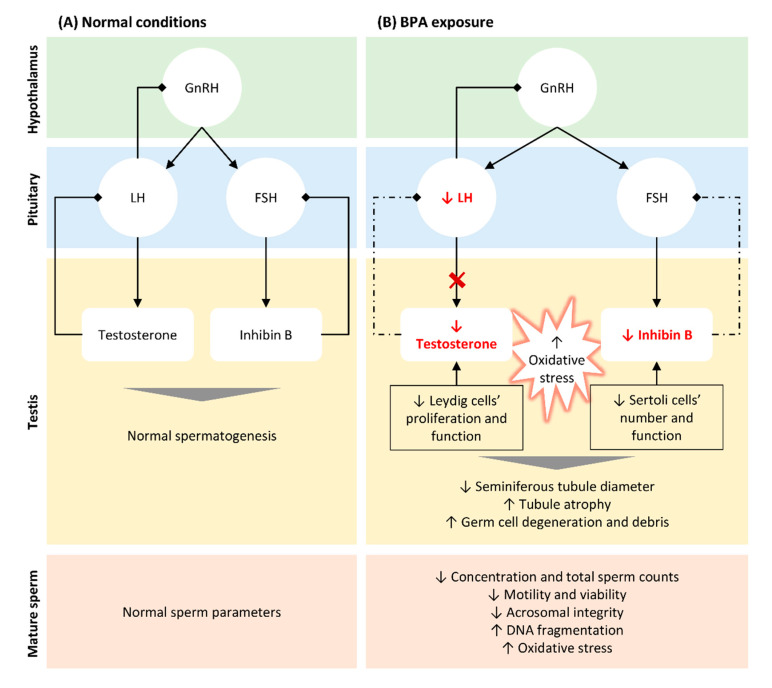
Schematic representation of BPA-induced alterations in hypothalamic–pituitary–testicular (HPT) axis, testicular function and structure, and in seminal parameters. (**A**). In normal conditions, gonadotrophin releasing hormone (GnRH) is released by the hypothalamus stimulating the secretion of follicle-stimulating hormone (FSH) and luteinizing hormone (LH) by the pituitary. LH acts on Leydig cells and FSH on Sertoli cells, stimulating the biosynthesis of testosterone and inhibin B, respectively. Both hormones are crucial for normal spermatogenesis and, thus, to produce normal sperm. When testosterone and inhibin are released in the bloodstream, they inhibit GnRH/LH and FSH secretion, respectively (negative feedback). (**B**). Even at low concentrations, BPA reduced the levels of testosterone by directly targeting Leydig cells, reducing their proliferation, and impairing normal steroidogenesis. Moreover, BPA indirectly suppresses the release of LH through aromatase upregulation in testis, blocking testosterone synthesis. The reduction of inhibin B observed following BPA exposure is also associated to a reduction in the number of Sertoli cells, directly affecting spermatogenesis. The lower levels of testosterone and inhibin B block the mechanism of negative feedback, in an attempt to increase the release of LH and FSH and their action in testis (dashed arrows). BPA exposure also results in an increase in free radicals, which associated with altered hormonal levels lead to histological alterations in testis and germ cells’ reduction and degenerations. These alterations explain the abnormal seminal parameters observed in situations of BPA exposure, such as decreased concentration and total sperm count, reduced motility and viability and increased DNA fragmentation.

The most significant risks associated with BPA exposure are attributed to its action as an EDC. It was shown that BPA has estrogenic activity deregulating the hypothalamic–pituitary–gonadal (HPG) axis even at low concentrations (Figure 1). Studies performed in animal models showed that BPA directly acts on Leydig cells, reducing their proliferation [32] and impairing the normal steroidogenesis by promoting (i) the production of 17- hydroxy-pregnenolone and testosterone from cholesterol, (ii) the expression of CYP19A1 that converts testosterone into E2, resulting in higher levels of the latter [69], and by reducing the expression of the steroidogenic enzyme 17α-hydroxylase/17–20 lyase [70]. Consistent with this finding, several in vitro and in vivo studies reported that BPA negatively affects testosterone production in both mice [71,72] and rat models [24,70,73,74], as well as in humans [14,73]. Moreover, BPA indirectly suppresses the synthesis and release of luteinizing hormone (LH) from the pituitary [70,71] through aromatase upregulation in testes, activating the mechanisms of negative hormonal feedback [71]. Additionally, human epidemiological studies showed that BPA modulates the levels of follicle stimulating hormone (FSH) [75,76], inhibin B [76,77], and E2 [76,77] in men. Interestingly, prenatal exposure to BPA resulted in abnormal foetal development and testicular endocrine function, associated with reduced Leydig cell proliferation and foetal testosterone production [72,73,74]. All these alterations result in impaired testosterone production, with consequent effects on spermatogenesis [78,79] (Figure 1).

Spermatogenesis is a highly complex process mainly regulated by testosterone and inhibin B, hormones released by Leydig and Sertoli cells, respectively [80]. Any disturbance in hormonal levels may compromise the spermatogenic process, resulting in abnormal semen parameters and reduced fertility. BPA disrupts spermatogenesis by inhibiting androgen production and reducing Sertoli cell number and function [30,81,82,83,84]. Furthermore, BPA exposure decreased the seminiferous tubule diameters and increased tubule atrophy and damage [26,27,85,86,87], induced germinal cell debris and congestion [27,86,88], as well as induced the reduction and/or degeneration of spermatocytes [25,27,32,89,90] and other spermatogenic cells [22,26,30,86,87,89,90]. A recent study in mice showed that chronic exposure to BPA impairs the proliferation of spermatogonia and spermatocytes, resulting in poor sperm quality, especially reduced sperm counts and motility [91]. In addition, these male mice exposed to BPA through drinking water for two months presented reduced serum testosterone levels, diminished pregnancy rates, and reduced fertilization efficacy compared with the non-exposed [91]. Interestingly, data from in vivo studies suggested that foetal BPA-associated endocrine disruption negatively impacts male fertility in adult life. Salian et al. reported that maternal exposure to BPA was associated with reduced sperm count and motility in F1 male offspring and their subsequent generations [92]. Moreover, a significant increase in post-implantation loss in BPA treated females and a decreased litter size in all generations was observed [92]. In mice, males exposed to BPA by oral ingestion presented reduced testes and seminal vesicles weight, with a consequent reduction in sperm count [93]. The diminished sperm count after BPA exposure was confirmed by several other studies in rodents [83,91,94,95,96,97,98,99] and humans [76,100]. Lower levels of exposure were also associated with reduced sperm motility [83,94,95,97,101,102] and acrosomal integrity [94], impaired markers of OS [83,94,95,97], and increased DNA fragmentation indexes [21,25,95,97,101,102,103,104] in animal models. In humans, several epidemiologic studies also reported a negative association between urinary BPA levels and sperm concentration, total sperm count [76], motility, and viability [31,76,79,105,106,107], but not with morphology [100]. The correlation between BPA exposure and alterations of sperm DNA was also observed in humans by evaluating the sperm DNA damage in a cohort of 190 subfertile male patients [108]. Surprisingly, the presence of this EDC in seminal fluid and how it correlates with semen quality were only reported in 2015 [14], requiring additional studies since the distribution and metabolism in this fluid are distinct from other biofluids [67]. Moreover, how BPA reaches the seminal fluid and how it impacts sperm maturation, for instance, during epididymal transit, is still unknown and deserves further investigation. Overall, it is now accepted that BPA affects the male reproductive system at several levels, disturbing steroidogenesis and spermatogenesis and resulting in poor fertility outcomes and reduced fertility in the progeny.

## 4. Impact of BPA Exposure on Oxidative Stress in Testis and Sperm

**Figure 2:**
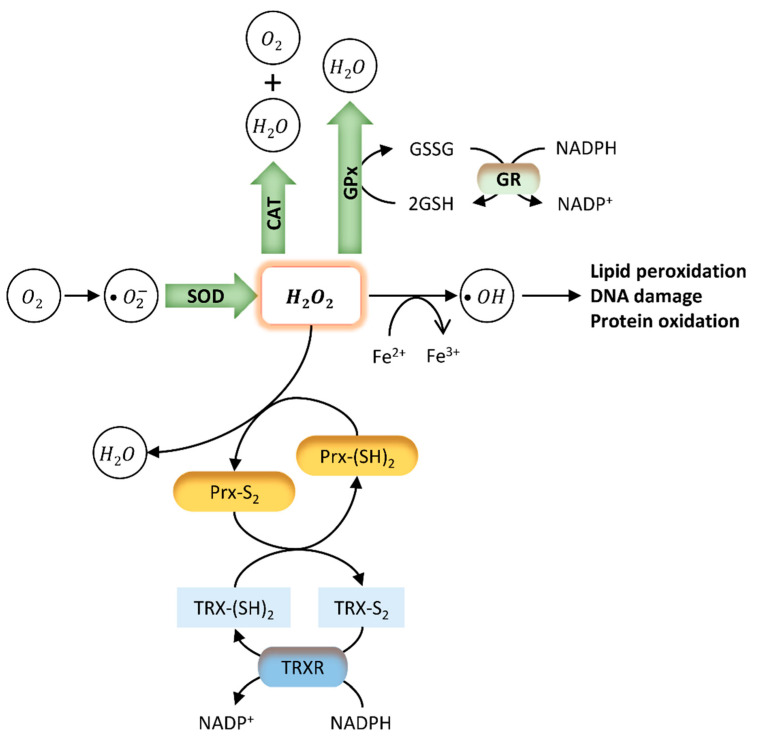
Endogenous antioxidant mechanisms. The oxidative stress (OS) caused by several external and internal factors leads to an excessive production of ROS intracellularly, including the free radicals hydroxyl (OH^−^), peroxyl (HO_2_), and superoxide (O_2_^−^). Cellular redox homeostasis is maintained by an endogenous antioxidant defence system that includes the endogenous antioxidant enzymes SOD, CAT, GPx, and GSH (green). These antioxidants directly scavenge O_2_^−^ and hydrogen peroxide (H_2_O_2_), converting them into less reactive species. SOD catalyses the dismutation of superoxide radical to H_2_O_2_. H_2_O_2_ is rapidly converted into OH^-^ radical, which is very reactive and causes lipid peroxidation and DNA damage. GPx neutralizes H_2_O_2_ by taking hydrogens from two GSH molecules resulting in two H_2_O and one GSSG. GR then regenerates GSH from GSSG. Finally, CAT neutralizes H_2_O_2_ into H_2_O and O_2_. Two other antioxidant systems involve peroxiredoxins (Prx) (yellow) and the thioredoxin (TRX) system (blue). Prx are ubiquitous antioxidant enzymes that catalyse the reduction of H_2_O_2_, peroxynitrite (ONOO^−^), and organic hydroperoxides to water, nitrite, or hydroxyl derivatives (ROH), respectively [118]. The TRX system composed by TRX, TRX reductase (TRXR), and NADPH is a ubiquitous thiol oxidoreductase system that also regulates cellular redox status [119]. Briefly, an initial oxidation of the active site of Prx forms an interchain disulphide (Prx-S2). The hyper-oxidation of Prx decreases localized peroxidase activity, leading to the oxidation of less sensitive proteins [118]. Reduced TRX (TRX-(SH)2) catalyses the reduction of disulphides (S-S) within oxidized proteins, including Prx - Prx-(SH)2. In this process, Trx becomes oxidized (TRX-S2), being further reduced by thioredoxin reductase (TRXR) at the expense of NADPH.

The imbalance between the excessive production of reactive oxygen species (ROS) and their neutralization and removal by the antioxidant system results in an increase in OS [109]. Cells present a complex system of antioxidant defences that contains antioxidant enzymes, molecular antioxidants, and metallic chemical agents, converting ROS into non-toxic forms [51]. Enzymatic antioxidants include superoxide dismutase (SOD), glutathione peroxidase (GPx), and catalase (CAT), which protect the living system from the harmful effects of ROS and reduce their oxidative damage to cell membranes [51,110] (Figure 2). SOD constitutes the first line of defence against superoxide radicals (O_2_^−^) by catalysing their dismutation to form hydrogen peroxide (H_2_O_2_) and oxygen (O_2_) [111]. H_2_O_2_ causes rapid and severe oxidative damage to lipids, proteins, and DNA [110]. Reduced SOD activity results in the accumulation of O_2_^−^, which in turn inhibits CAT activity, decreasing the cells ability to eliminate H_2_O_2_ [51]. On the other hand, GPx may act directly as an antioxidant enzyme, catalysing the reduction of phospholipid hydroperoxides within membranes and lipoproteins [112]. Since high ROS levels promote oxidation of biomolecules such as nucleic acids, lipids, and proteins, these enzymes constitute important intracellular antioxidants to protect against ROS-mediated damage [111,113]. Molecular antioxidants are typically scavenging/non-enzymatic antioxidants (NADPH, glutathione, vitamins, flavonoids, carotenoids, melatonin) that bind to active free radicals and disrupt chain propagation reactions [51]. These antioxidants donate an electron to free radicals to neutralize them, becoming free radicals with reduced toxicity that are easily neutralized by other antioxidants in the same class. Finally, some metals, such as zinc (Zn) have important antioxidant and anti-inflammatory properties [114,115]. Zinc, copper (Cu), and iron (Fe) are essential components of the antioxidant enzymes Cu-SOD, Zn-SOD, and CAT, respectively [116]. Indeed, it was reported that Zn deficiency aggravates the toxicity of BPA in rat testis, increasing cellular and DNA damage, apoptosis, and modifying protein expression [117].

Testicular structural damage and dysfunction are often associated with increased oxidative stress; however, in spermatozoa, small amounts of ROS are required for specific and essential functions, such as capacitation [120], acrosome reaction [121], and motility hyperactivation [122]. Moreover, spermatozoa produce small amounts of ROS as a by-product of the electron transfer chain in mitochondria [109,120]. However, increased ROS levels can induce errors during DNA replication, transcription or post-transcriptional events, resulting in sperm DNA fragmentation, chromatin condensation abnormalities, and protamine expression defects [123]. In fact, OS is considered the principal cause of DNA damage in spermatozoa [124,125,126].

Several conditions may increase ROS production in testis and sperm, like varicocele or infections, as well as environmental factors or lifestyle (smoking, alcoholism, medication, radiation). Numerous in vitro and in vivo studies reported that BPA, like most environmental contaminants, can induce testicular damage and consequently impaired semen parameters by inducing OS [105,106,127,128]. The in vitro exposure of *Acipenser ruthenus* spermatozoa to concentrations of BPA possibly occurring in nature (0.5–10 µg/kg) resulted in a significant reduction in sperm motility and velocity and an increase in DNA fragmentation, together with higher levels of protein and lipid oxidation and increased SOD activity [23]. Indeed, it was estimated that at least for humans, the range of exposure to BPA is between 0.4 and 5 ug/kg/day. More recently, using an in vitro experimental model, Rahman and colleagues showed that mice spermatozoa exposed to 100 µM BPA for 6 h have a significant decrease in the percentage of motile spermatozoa and intracellular ATP levels, increased activity of protein kinase-A (PKA), tyrosine phosphorylation and ROS levels [129]. These results are supported by the study of Rezaee-Tazangi et al. that reported that isolated mice testicular mitochondria treated with 800 µM BPA have significantly higher levels of ROS, malondialdehyde (MDA), and mitochondrial membrane potential (MMP) than the control, as well as reduced SOD and glutathione (GSH) levels [130]. Moreover, BPA considerably impaired epididymal sperm motility and viability, possibly due to the triggering of OS [130]. Indeed, in vitro exposure of human spermatozoa to BPA resulted in mitochondrial dysfunction (decreased MMP and increased mitochondrial generation of O_2_^−^) with a consequent reduction in sperm motility and increased DNA oxidative damage [106]. Following 4 h of exposure, the levels of caspase-3 and caspase-9 activation also increased, explaining the reduced sperm vitality observed [106]. In addition, the increased testicular ROS production may result in mitochondria dysfunction in Sertoli cells, leading to apoptosis [82]. Primary Sertoli cell cultures exposed to intermediate doses of BPA (10 and 50 μM) showed increased GSH content due to increased GSH synthesis and recycling enzyme expression without affecting cell viability [81]. However, 100 μM of BPA are deleterious for Sertoli cells, indicating a dose-response of Sertoli cells to BPA. However, most of the described in vitro experiments used BPA concentrations several times higher than what naturally occurs. For instance, the concentrations used in in vitro experiments using spermatozoa range from 10 to 800 µM, a much higher concentration than the maximum found in the seminal fluid (10.9 µg/L = 0.048 µM) [67], and thus, that reach spermatozoa in vivo. This type of studies may be interesting to study the immediate effects of an acute exposure to high levels of BPA; however, whether the concentration and the exposure time used demonstrate what happens at the physiological level and have biological relevance remains questionable.

Studies from mice revealed that in vivo exposure to BPA decreased testicular activities of the mitochondrial enzymes succinate dehydrogenase (SDH), malate dehydrogenase (MDH), isocitrate dehydrogenase (IDH), monoamine oxidase (MAO), and NADH dehydrogenase (NDH) [21]. It also affects the activity of antioxidant enzymes, reducing the activity of SOD [21,25,131], glutathione reductase (GR) [21], CAT [131], and GPx [21,22]. Additionally, results showed that BPA caused LPO [21,22] and decrease GSH content in mitochondria [21,131]. In rats, it was also found that BPA deregulate not only testosterone levels and semen quality but also induced OS in testis and epididymal sperm, by increasing the levels of MDA [26,27,30,32,86,88], H_2_O_2_ [26,86,102,105,132], and LPO [105,132] and decreasing the GSH content [26,27,30,86,88] and CAT [26,88,105,132], SOD [25,26,30,86,88,105,132], GR [105], and GPx activity [26,30,86,88,105,132]. Increased MDA and decreased GSH concentrations are usually associated to higher concentrations of free oxygen radicals, inducing LPO in tissues. 

In an attempt to elucidate the signalling mechanisms underlying BPA-associated OS damage in testis, Yin and colleagues expose mouse spermatocytes GC-2 cells and adult mice to BPA [133]. The authors reported that in both models, BPA exposure induced not only mitochondrial damage but also endoplasmic reticulum (ER) injury, upregulating ER stress-related proteins (GRP78, p-PERK, p-EIF2α, chop and ATF6) in mice testis and GC-2 cells [133]. By inhibiting the PERK/EIF2α/CHOP branch of the ER unfolded protein response (UPR^ER^), the BPA-induced apoptosis observed both in vitro and in vivo was attenuated [133], suggesting that the BPA-induced male reproductive toxicity results, at least in part, from the activation of PERK/EIF2α/chop pathway in response to the elevation of ROS levels. Altogether, these findings support that BPA-induced testicular damage, and abnormal semen parameters are in part associated with an increase in OS caused by the elevated production of ROS and a deficient antioxidant system. Moreover, BPA exposure induced testicular mitochondrial damage and LPO, as well as ER stress, activating stress response-related signalling pathways. Despite the valuable data obtained by studying animal models, evidence in humans is still scarce and weak, and efforts to understand how these results can be transposed to the clinic should be implemented.

## 5. Ameliorative Effects of Antioxidants in BPA-Induced Reproductive Toxicity

In the past years, several research groups have focused their investigation on possible approaches to treat or prevent BPA-induced testicular toxicity and male infertility. Since the effect of BPA on testicular cells and mature spermatozoa are particularly due to OS, most of the pharmacological approaches are based on the use of compounds with antioxidant properties (Table 1). Antioxidants are reducing agents capable of scavenge and neutralize free radicals, inhibiting oxidation and preventing OS in cells and tissues.

### 5.1. Melatonin

The neurohormone melatonin (*N*-acetyl-5-methoxytryptamine), a free radical scavenger with a significant antioxidant activity [136], increases the levels and activity of the antioxidant enzymes SOD [137], GPx and GR [138]. It also reduces mitochondrial LPO [138], a feature often observed in testis after BPA exposure [21]. 

Several studies investigated the role of melatonin in ameliorating BPA-induced testicular toxicity [21,25,26,85]. Anjum et al. showed that the oral administration of melatonin in mice exposed to BPA reduced testicular mitochondrial LPO; restored the activity of mitochondrial enzymes (SDH, MDH, IDH, MAO, and NDH); and improved the mitochondrial antioxidants GPx, SOD, and GR [21]. In rat testes and epididymal sperm, similar results were reported—the administration of melatonin with BPA [26] and BPA exposure in utero [86] decreased OS by restoring GSH, GPx, SOD, CAT, and Glutathione-S-transferase (GST) activity and MDA and H_2_O_2_ levels. This potent antioxidant also normalizes testosterone levels, improves histopathological alterations and allows the occurrence of normal spermatogenesis, leading to normal sperm count, motility, viability, and morphology [26,86]. By studying the genotoxic effects of BPA in male Sprague Dawley rats’ germ cells and the potential protective action of melatonin, Wu and colleagues showed that animals exposed to 200 mg/kg BPA per day for ten days presented higher levels of thiobarbituric acid reactive substances (TBARS) and decreased activity of SOD than controls [25]. Additionally, they reported increased DNA damage at pachytene spermatocytes stage in rats exposed to BPA, accompanied by an increased frequency of γH2AX foci, a marker of double strand breaks [25]. The authors also observed that the detected effects were significantly alleviated by melatonin pre-treatment [25]. Altogether, these data suggest that BPA induce DNA damage accumulation in the germ cells of rats via OS, which can be effectively prevented by melatonin. Moreover, melatonin supplementation has the potential to protect male fertility and assist normal fertilization, by preventing the transference of defective paternal DNA to the offspring.

Recently, it was reported that melatonin not only protects rat testis and sperm against BPA damage, but also the epididymis [85]. The histological alterations in rat epididymis resultant from the exposure to BPA diminished, and the seminal quality improved with melatonin administration [85]. Additionally, BPA reduced the levels of claudin-1 (Cldn-1), occluding (Occ) and zonula occludens (ZO-1), components of the blood–epididymis barrier (BEB) that are critical for the integrity of tight junctions, which is ameliorated by melatonin administration [85]. These data support the vision that melatonin prevents the BPA-induced disruption of BEB that leads to changes in sperm maturation and thus to altered sperm motility and viability.

### 5.2. Vitamins

In rats, supplementation with folic acid (Vitamin B9), well known for its antioxidant properties, also seems to minimize testicular toxicity induced by BPA [134]. According to Gules’ results, the group exposed to folic acid followed by BPA have improved serum testosterone levels, sperm viability and morphology, and reduced apoptosis, compared with the BPA treated group, and similar phenotype than the control group [134]. On the other hand, the co-administration of the dietary antioxidant vitamin C with BPA did not seem to result in any benefit on testicular BPA-induced oxidative damage in rats [27]. Indeed, the increase in MDA and morphologically abnormal sperm, and the decrease in GSH levels observed in BPA treated group were also observed in the vitamin C co-administrated group [27]. Besides, in the vitamin C + BPA group, aggravated histological alterations were observed compared with BPA treated group (increased atrophy and germ cells debris), which may be associated with the pro-oxidant properties of vitamin C [27]. In sperm, in vitro experiments using a mice model showed that the administration of vitamin C, vitamin E and GSH effectively prevent BPA-induced OS [129]. The authors reported that these antioxidants inhibit the excessive production of ROS and increase intracellular ATP, avoiding motility loss caused by exposure to BPA [129]. Additionally, vitamin E and GSH reduced tyrosine phosphorylation in sperm, preventing premature abnormal acrosome reaction [129]. Interestingly, the preventive effects of vitamin E and GSH are more evident than those from vitamin C since the administration of vitamin C resulted in an incomplete recovery of the damages [129]. Additionally, vitamin A also seems to restore sperm motility and normal morphology in mice exposed neonatally to BPA [135]. Aikawa et al. reported that mice exposed to 50 µg of BPA during the first 5 days after birth presented diminished sperm motility and increased morphologically abnormal spermatozoa, which are ameliorated by retinol acetate administration, a naturally occurring metabolite of vitamin A [135]. However, the limited number of studies available question the reliability of these evidence, requiring more investigation to clearly elucidate the role of vitamins in fertility protection against BPA. 

### 5.3. Natural Extracts

Recently, several phytochemicals [88,89] and plant extracts [30,31,32,90] showed an ameliorative effect on testicular function and semen quality in human and animal models exposed to BPA. *Cordyceps militaris*, a medicinal fungus widely used in traditional Chinese medicine, contains many active components, such as cordycepin, polysaccharides, and cordycepin acid, with anti-bacterial, anti-tumour and anti-oxidative properties [139]. Experimental evidences showed that this fungus’ extract restored the histological architecture of seminiferous tubules and epididymis and improved sperm count and motility in male rats exposed to BPA through OS reduction [30]. In particular, the administration of *C. militaris* significantly increased testicular SOD, GPx, and GSH, as well as reduced serum MDA, inhibiting LPO [30]. Additionally, it restored the serum concentration of LH and testosterone, reduced by BPA administration, increasing the expression of key players in steroidogenesis (StAR, CYP11A1, 3β-HSD, and CYP17A1) [30]. Two other compounds used in Chinese medicine—*Cistanche tubulosa* and echinacoside—were also shown to have the potential to protect testis and sperm against BPA injury. Echinacoside, the major active ingredient of *Cistanche tubulosa*, has several health benefits including anti-inflammatory, antioxidant, and neuroprotective characteristics [140]. Similar to *C. militaris,* the use of these compounds reversed BPA-induced abnormalities in rat sperm, testicular structure, and serum testosterone levels by enhancing StAR, CYP11A1, 3β-HSD, 17β-HSD, and CYP17A1 levels [87], making them a promising natural resource to develop therapeutic agents. Additionally, the herbal medicines *Trigonella foenum-graecum* (Fenugreek) and *Lespedeza cuneata* also have potent antioxidant properties, improving sperm parameters, testis weight and histoarchitecture, testosterone levels, and the levels of antioxidant enzymes in BPA-treated mice [90,131]. In vitro co-treatment of TM4 Sertoli cells with BPA and 50, 100, or 200 µg/mL *Lespedeza cuneata* ethanol extract for 24h also recovered cell viability by attenuating Bax expression and inactivating caspase 3 and PARP [131]. This extract, extremely enriched in bioactive substances such as β-sitosterol, quercetin, kaempferol, pinitol, avicularin, juglanin, trifolin, vitamins, and flavonoids, has the potential to protect male reproductive health against BPA injury. 

By studying the protective effect of *Eruca sativa* aqueous extract (ESAE) in disturbances induced by BPA in vitro, Grami et al. showed that human sperm exposed to BPA presented reduced motility and viability and diminished MMP [31]. Even at low doses, this medicinal plant extremely rich in natural antioxidants, such as polyphenols (gallic acid) and flavonoids (quercetin, kaempferol, cirsilineol, and acacetin), protects against BPA toxicity both in vitro and in vivo [31,32]. In vivo experiments showed that *Eruca sativa* aqueous extract supplementation significantly restores the activity of antioxidant enzymes such as SOD, CAT, and GPx in rat testis and epididymis subjected to BPA treatment [32]. However, the treatment with higher concentrations was associated with a severe mitochondrial dysfunction and cell membrane redox balance, decreasing sperm motility [31,32]. Thus, studies should be performed to establish the appropriate dose, avoiding cumulative toxic effects. 

The therapeutic effects of several phytochemicals (flavonoids, lycopene) against BPA-induced reproductive toxicity have also been investigated. Naringin is a flavone found in citrus, tomatoes, cherries, grapefruit, and cocoa that presents several functions, such anti-oxidative, anti-cancer, and anti-inflammatory activities [141]. Wistar rats exposed to BPA were treated with naringin, presenting normal levels of serum hormones (LH, FSH, testosterone, and E2), improved sperm counts and testicular histology, and a better antioxidant system [88]. Furthermore, the co-administration of BPA and quercetin (3, 5, 7, 3ʹ,4ʹ-pentahydroxyflavone) reversed the toxic effects of BPA on testis and epididymis of Sprague Dawley rats, restoring spermatogenesis, histopathological damages, and lipid profile [89]. Moreover, the powerful antioxidant lycopene, a natural carotenoid present in tomato and tomato products, showed a positive effect in vivo, by protecting rat testis from germ cells’ loss, preventing testis and epididymis loss of weight and restoring the impairment of sperm motility by normalizing the activity on anti-oxidant enzymes [132]. Thus, the protective effects of lycopene against BPA- induced abnormal sperm rates, and OS may be attributed to its anti-lipid peroxidative and free radical scavenging properties. Despite promising the use of exogenous antioxidants may be carefully used due to possible adverse effects.

### 5.4. Other Antioxidants

Recently, Rezaee-Tazangi and colleagues investigated the in vitro effects of taurine (TAU) on BPA-induced OS in testicular mitochondria and on sperm viability and motility [130]. TAU (2-aminoethanesulfonic acid) is a free amino acid present in several tissues that may act as an antioxidant in sperm. It was shown that pre-treatment with TAU suppressed BPA-induced mitochondrial OS, enhanced MMP and improved sperm viability and motility in a dose-dependent manner [130]. Interestingly, studies performed in other tissues, such heart, showed that the decrease in GPx, SOD, GST, and CAT activities in BPA exposed groups were not reverted by the administration of TAU or curcumin [142], which suggest a tissue/cell-dependent response. The protective effect of Selenium (Se) against BPA-induced reproductive toxicity in male mice and rats was also reported [22,143]. This vital micronutrient seems to prevent testicular damage by decreasing LPO and OS in testes, resulting in lower apoptotic index of germ cells and improved semen parameters, compared to BPA-treated animals [22,143]. Several studies also demonstrated that BPA-induced OS can lead to changes in DNA methylation levels in testis, and supplementation with antioxidants, including N-acetylcysteine (NAC), was able to restore these changes by improving the antioxidant system [28,101]. Minamiyama et al. (2010) reported that decreased sperm motility and increased ROS levels associated with BPA exposure were reversed by the administration of NAC prior to the exposure to BPA in male rats [101]. However, NAC did not always exhibit a protective effect, since higher concentrations exacerbate OS [28].

## 6. Conclusions

BPA is now recognized as a potent endocrine disruptor that compromises the HPG axis during foetal and adult life and disturbs the cellular redox balance in testis and sperm, resulting in altered testis development, architecture and function, impaired endocrine function, and abnormal semen parameters. Overall, available data support an adverse effect of BPA on sperm characteristics, such as reduced motility and concentration, and increased genetic abnormalities; however, these alterations were not accompanied by clear data on fertility outcomes. At the molecular level, increased ROS production, mitochondrial dysfunction, and redox imbalance seem to be important factors for BPA-induced testicular damage.

The recognition of effective markers of exposure able to determine and predict the health and reproductive consequences and the identification of therapeutic moieties capable of rescue the BPA-induced toxicity on the male reproductive system represent the major challenges in this field. Antioxidants that reduce OS, lipid peroxidation, and DNA damage, restoring the global antioxidant defence system, can be used to treat male infertility and poor semen quality associated with BPA exposure. Many antioxidants such as Vitamin E, melatonin, and *N*-acetylcysteine seem to have potential benefits to ameliorate BPA-induced toxicity, in vitro and in vivo. However, this supplementation for prevention/treatment of altered states associated with environmental exposure in humans should be considered with caution, considering that the available studies are limited and were performed in animal models, and further studies are required to establish the appropriate dosage and treatment scheme. Additional research should also be conducted to confirm the safety and efficacy of these antioxidants for its clinical application.

## Figures and Tables

**Table 1:** Antioxidants used to treat or prevent BPA-induced male fertility and their effects. The animal model used in each study, the experimental design, and the effects of the coadministration of BPA and antioxidant compared with the effects of BPA exposure alone were also presented.

Reference	Animal	Antioxidant	Experimental Groups (G)	Effects of BPA + Antioxidant Administration
[21]	Swiss albino mice (in vivo)	Melatonin (hormone)	G1: 0.2 mL olive oil (control); G2: 10 mg/kg BPA suspended in olive oil; G3: 10 mg/kg melatonin; G4: BPA 10 mg/kg + 10 mg/kg of melatonin—dose/day for 14 days	↑ Mitochondrial marker enzymes SDH, MDH, IDH, NDH, MAO, GSH, antioxidant enzymes GPx, SOD, GR ↓ LPO
[25]	Sprague Dawley rats (in vivo)	Melatonin (hormone)	G1: 0.5% ethanol in normal saline (control); G2: 200 mg/kg BPA suspended in olive oil; G3: 10 mg/kg melatonin intraperitoneally 30 min before BPA administration; G4: 10 mg/kg melatonin intraperitoneally + 200 mg/kg BPA suspended in olive oil—dose/day for 10 days	↔ body weight, reproductive organs weight, testes/body and epididymis/body weight ratios, sperm counts and apoptosis ↑ SOD activity and 4C-cells number ↓ TBARS accumulation and DNA damage in spermatocytes, number of γH2AX-positive foci
[26]	Sprague Dawley rats (in vivo)	Melatonin (hormone)	G1: no treatment (normal control); G2: 0.2 mL corn oil (experimental control); G3: normal saline (experimental control); G4: 50 mg/kg BPA suspended in corn oil; G5: 10 mg/kg melatonin in normal saline; G6: 10 mg/kg melatonin + 50 mg/kg BPA—3 days/week for 6 weeks	↑ sperm count and motility, testosterone levels, GSH, viable cells ↓ mortality and abnormal sperm, % diploid sperm and spermatid; levels of H_2_O_2_ and MDA, necrotic and apoptotic cells Other alterations: seminiferous tubules showed increase in the germinal cell population with active spermatogenesis and normal arrangement of spermatogenic cell, Leydig cells population normal
[85]	Sprague Dawley rats (in vivo)	Melatonin (hormone)	G1: 25 mg/kg sesame oil + 25 mg/kg 0.1% ethanol (control); G2: 25 mg/kg BPA; G3: 25 mg/kg BPA + 20 mg/kg melatonin—dose/day for 60 days	↔ total sperm counts ↑ Cldn-1, Occ and ZO-1 immunostaining, sperm motility Other alterations: Fewer vacuolations, irregular tubules and degenerative cells containing a heterochromatic nucleus in epididymis
[86]	Wistar albino rats (in vivo)	Melatonin (hormone)	G1: 0.2 mL 1% dimethyl sulfoxide (DMSO)/99% canola oil (control); G2: 0.025 mg/kg BPA; G3: 0.25 mg/kg BPA; G4: 0.025 mg/kg BPA + melatonin 1 mg/kg; G5: 0.25 mg/kg BPA + melatonin 1 mg/kg—dose/day; exposure in utero from gestational day 10–21	↑ body weight; gonosomatic index; sperm motility; viability and count; serum T levels and LH; activity of SOD, GSH, GPx, and GST; tubular and luminal diameter ↓ FSH and E2; testicular MDA and H_2_O_2_ levels, interstitial necrosis, and germinal cell degeneration
[134]	Wistar albino rats (in vivo)	Folic acid (vitamin B9)	G1: 0.5 mL 0.9% NaCl (control); G2: 50 mg/kg BPA in 0.5 mL corn oil; G3: 20 mg/kg/day folic acid in 0.5 mL 0.9% NaCl; G4: 20 mg/kg folic acid in 0.5 mL 0.9% NaCl + 50 mg/kg BPA in 0.5 mL corn oil—dose/day for 14 days	↔ body weight, testes/body weight ratios, number of UTF-1 positive cells/tubule and UTF-1 positive tubules ↑ serum testosterone levels, viable sperm ↓ TUNEL positive cells and tubules, head, midpiece and total sperm abnormalities
[27]	Wistar albino rats (in vivo)	Vitamin C	G1: olive oil (control); G2: 25 mg/kg/day BPA; G3: 25 mg/kg/day BPA + 60 mg/kg/day of vitamin C three times a week—50 days	↑ right epididymal weight, congestion areas, atrophy, germinal cell debris ↓ GSH
[129]	CD-1 (ICR) mice (in vitro)	Vitamin C, Vitamin E and GSH	Condition I: DMSO (control); Cond II: 100 µM BPA; Cond III: 100 µM BPA + 5 mM GSH; Cond IV: 100 µM BPA + 100 µM Vitamin C; Cond III: 100 µM BPA + 2 mM of Vitamin E—for 6 h	↑ sperm motility, ATP levels ↓ acrosome-reacted spermatozoa, PKA activity, protein tyrosine phosphorylation and nitration, ROS levels
[135]	SHN mice (in vivo)	Vitamin A	G1: 16 mL of sesame oil and 4 mL of dimethyl sulfoxide (control); G2: 0.5 mg BPA; G3: 50 mg BPA; G4: 50 mg BPA + 100 IU Retinoic Acid—for 5 days from the date of birth	↑ sperm motility ↓ abnormal sperm
[31]	Human (in vitro)	*Eruca Sativa* aqueous extract	Condition I: untreated (control); Cond II: 10 µM BPA; Cond III: 10 µM BPA + 15.5 µg/mL ESAE; Cond IV: 10 µM BPA + 62.55 µg/mL ESAE; Cond V: 10 µM BPA + 250 µg/mL ESAE; Cond VI: 10 µM BPA + 1000 µg/mL ESAE—ESAE incubation for 1 h followed by BPA incubation for 4 h	↑ sperm progressive motility and viability, mitochondrial function ↓ immotile sperm
[32]	Wistar albino rats (in vivo)	*Eruca Sativa* aqueous extract	G1: 0.4 mL/kg/day of tocopherol-stripped corn oil (control); G2: 100 mg/kg BPA; G3: 200 mg/kg ESAE; G4: 100 mg/kg BPA + 50 mg/kg ESAE; G5: 100 mg/kg BPA + 100 mg/kg ESAE; G6: 100 mg/kg BPA + 200 mg/kg ESAE—dose/day for 30 days	↑ body weight, reproductive organs weight, testosterone, and LH levels, sperm counts, motility, viability, SH group content ↓ morphologically abnormal sperm; MDA levels; SOD, CAT and GPx activities
[30]	Sprague Dawley rats (in vivo)	*Cordyceps militaris*	G1: no intervention (normal control); G2: 200 mg/kg BPA; G3: 800 mg/kg *C.militaris*; G4: 200 mg/kg BPA + 200 mg/kg *C. militaris*; G5: 200 mg/kg BPA + 400 mg/kg *C. militaris*; G6: 200 mg/kg BPA + 800 mg/kg *C. militaris*; G7: 200 mg/kg BPA + 300 mg/kg Vitamin E—28 days	↑ body weight; SOD, GPx, GSH, testosterone, and LH serum levels; sperm counts and motility; mRNA levels of Star; CYP11A1; 3β-HSD; and CYP17A1 ↓ MDA levels
[87]	Sprague Dawley rats (in vivo)	*Cistanche tubulosa* and Echinacoside (ECH)	G1: corn oil 10 mL/kg (normal control); G2: 200 mg/kg BPA; G3: 200 mg/kg BPA + 300 mg/kg Vitamin E; G4: 200 mg/kg BPA + 6 mg/kg ECH; G5: 200 mg/kg BPA + 200 mg/kg CT; G6: 6 mg/kg EC; G7: 200 mg/kg CT—6 weeks	↑ sperm motility; LDH-x activity; FSH, LH, and testosterone serum levels; mRNA levels of StAR, CYP17A1, 3β-HSD, and 17β-HSD; protein levels of CYP11A1 and CYP17A1 ↓ abnormal sperm Other alterations: normal histological pattern, normal spermatogenic series
[88]	Wistar albino rats (in vivo)	Naringin (flavonoid)	G1: Control; G2: 50 mg/kg BPA; G3: 50 mg/kg BPA + 40 mg/kg naringin; G4: 50 mg/kg BPA + 80 mg/kg naringin; G5: 50 mg/kg BPA + 160 mg/kg naringin; G6: 160 mg/kg Naringin—for 30 days	↔ body weight ↑ testicular weight and volume; total testicular protein; epididymal sperm count; testicular enzymes (ALP, LDH); serum FSH; LH; testosterone and E2; activities of GPx, SOD, and CAT; GSH ↓ MDA, ROS Other: less testicular tissue damage
[89]	Sprague Dawley rats (in vivo)	Quercetin (flavonoid)	G1: normal saline (control); G2: 50 mg/kg BPA; G3: 50 mg/kg quercetin; G4: 50 mg/kg BPA + 50 mg/kg quercetin—for 52 days	↔ body weight, reproductive organ weight ↑ plasma testosterone, LDL and HDL levels, tunica albuginea thickness, seminiferous tubule area, number of spermatogonia, primary spermatocytes, secondary spermatocytes, and spermatids ↓ oestrogen levels, blood urea nitrogen levels, creatinine, cholesterol, triglyceride levels
[90]	Balb/c mice (in vivo)	*Trigonella foenum-graecum*	G1: normal pellet diet (control); G2: 200 mg/kg fenugreek seeds aqueous extract; G3: 1 mg/kg BPA; G4: 1 mg/kg BPA + 200 mg/kg fenugreek seeds aqueous extract—2 months	↑ testis weight, sperm concentration, sperm motility, GSH, GPx activity, Bcl-2 mRNA levels ↓ ROS and LPO, Caspase-9 and -3 mRNA level Other alterations: improved histoarchitecture, basement membrane preservation with less vacuolization and increased number of elongated, round spermatids
[131]	CD-1 (ICR) mice (in vivo)	*Lespedeza cuneata* ethanol extract (LCE)	G1: normal saline (solvent control); G2:10 mg/kg BPA; G3: 10 mg/kg BPA + 100mg/kg *Saw Palmetto* extract (SPE); G4: 10 mg/kg BPA + 25 mg/kg LCE; G5: 10 mg/kg BPA + 50 mg/kg LCE; G6: 10 mg/kg BPA + 100 mg/kg LCE—for 12 weeks	↑ testis weight; sperm counts and motility; testosterone levels; GSH, CAT, and SOD1 levels; HDL-cholesterol ↓ sperm abnormalities; TBARS levels; glucose; TC, TG, and LDL- cholesterol
[132]	Sprague Dawley rats (in vivo)	Lycopene (carotenoid)	G1: saline following treatment with 0.5 mL corn oi (control); G2: 200 mg/kg BPA; G3: 200 mg/kg BPA + 10 mg/kg lycopene; G4: 10 mg/kg lycopene—for 30 days	↑ body and organ weight, sperm count, sperm motility, antioxidants enzymes level (SOD, CAT, GPx, GR) ↓ LPO and H_2_O_2_
[130]	NMRI mice (in vitro)	Taurine (amino acid)	Condition I: untreated (control); Cond II: 0.8 mmol/L BPA for 2 h; Cond III: 50 µmol/ L TAU for 4 h; Cond IV: pre-treated with 5 µmol/L of TAU for 2 h before BPA treatment (2 h); Cond V: pre-treated with 10 µmol/L of TAU for 2 h before BPA treatment (2 h); Cond VI: pre-treated with 30 µmol/L of TAU for 2 h before BPA treatment (2 h); Cond VII: pre-treated with 50 µmol/L of TAU for 2 h before BPA treatment (2 h)	↑ Sperm and testicular mitochondria viability, MMP, GSH, SOD, sperm motility ↓ testicular mitochondrial ROS, MDA
[22]	BALB/c mice (in vivo)	Selenium	G1: diet adequate in selenium (0.2 ppm/kg diet) as sodium selenite for 12 weeks (control); G2: 0.5 ppm sodium selenite/kg for 12 weeks; G3: 0.2 ppm sodium selenite/kg for 8 weeks followed by 1 mg/kg BPA for 4 weeks; G4: 0.5 ppm sodium selenite/kg for 8 weeks followed by 1 mg/kg BPA for 4 weeks	↑ sperm concentration and motility, GPx activity ↓ ROS and LPO levels, number of TUNEL- positive germ cells Other alterations: preserved basement membrane with less vacuolization, increased germ cell count
[28]	*Gobiocypris rarus* (in vivo)	NAC	G1: 0.001%DMSO (control); G2: 10 mg/kg NAC; G3: 100 mg/kg NAC; G4: 225 μg/L BPA; G5: 10 mg/kg NAC + 225 μg/L BPA; G6: 100 mg/kg NAC + 225 μg/L BPA — for 7 days	↑ GPx activity ↓ levels of 5-methylcytosine (5mC), GSH, γ-glutamyl cysteine synthetase (GCS), DNA methyltransferase proteins (DNMTs), H_2_O_2_ concentration, S-adenosylhomocysteine (SAH), homocysteine (HCY), nicotinamide adenine dinucleotide phosphate (NADPH) levels, SOD, CAT activities
[101]	Wistar albino rats (in vivo)	NAC	0, 1.0 or 10 mg/L BPA for 8 weeks and BPA + 0.45% NAC for 2 days prior to the administration of BPA	↑ sperm motility ↓ HNE-modified protein at 30 kDa, ROS levels

Legend: ↔ no change; ↑ increase; ↓ decrease.
